# Enhanced analysis of the genomic diversity of *Mycobacterium bovis* in Great Britain to aid control of bovine tuberculosis

**DOI:** 10.3389/fmicb.2025.1515906

**Published:** 2025-03-25

**Authors:** Prizam Sandhu, Javier Nunez-Garcia, Stefan Berg, Jo Wheeler, James Dale, Paul Upton, Jane Gibbens, R. Glyn Hewinson, Sara H. Downs, Richard J. Ellis, Eleftheria Palkopoulou

**Affiliations:** ^1^Department of Bacteriology, Animal and Plant Health Agency, Addlestone, United Kingdom; ^2^Field Epidemiology, Animal Health and Welfare Advice Team, Professional Advice and Standards Directorate, Animal and Plant Health Agency, Addlestone, United Kingdom; ^3^Department of Epidemiological Sciences, Animal and Plant Health Agency, Addlestone, United Kingdom; ^4^Consultant Veterinary Epidemiologist, London, United Kingdom; ^5^Sêr Cymru Centre of Excellence for Bovine TB, Aberystwyth University, Aberystwyth, United Kingdom; ^6^Department of Surveillance and Laboratory Services, Animal and Plant Health Agency, Addlestone, United Kingdom

**Keywords:** *Mycobacterium bovis*, whole genome sequencing, bovine tuberculosis (bTB), epidemiology, phylogenetics, clade

## Abstract

Bovine tuberculosis (bTB) is an endemic disease in Great Britain (GB) that affects mainly cattle but also other livestock and wild mammal species, leading to significant economic and social impact. Traditional genotyping of *Mycobacterium bovis* (*M. bovis*) isolates, which cause bTB, had been used routinely since the late 1990s as the main resource of genetic information in GB to describe their population and to understand their epidemiology. Since 2017, whole-genome sequencing (WGS) has been implemented on *M. bovis* isolates collected during routine surveillance. In this study, we analysed genome sequences from 3,052 *M. bovis* isolates from across GB to characterise their diversity and population structure in more detail. Our findings show that the *M. bovis* population in GB, based on WGS, is more diverse than previously indicated by traditional genotyping and can be divided into seven major clades, with one of them subdivided further into 29 clades that differ from each other by at least 70 single-nucleotide polymorphisms (SNPs). Based on the observed phylogenetic structure, we present a SNP-based classification system that replaces the genotype scheme that had been used until recently in GB. The predicted function and associated processes of the genes harbouring these SNPs are discussed with potential implications for phenotypic/functional differences between the identified clades. At the local scale, we show that WGS provides greater discriminatory power and that it can reveal the origin of infection and associated risk pathways even in areas of high bTB prevalence. The difficulty in determining transmission pathways due to the limited discrimination of isolates by traditional typing methods has compromised bTB control, as without such information it is harder to determine the relative efficacy of potential intervention measures. This study demonstrates that the higher resolution provided by WGS data can improve determination of infection sources and transmission pathways, provide important insights that will inform and shape bTB control policies in GB, as well as improve farm specific advice on interventions that are likely to be effective.

## 1 Introduction

Bovine tuberculosis (bTB) is one of the most significant animal health challenges in Great Britain (GB) that impacts the livestock sector and results in considerable costs to the government and industry, and has major implications for farmers (Godfray et al., 2018). Despite long-standing control strategies including test-and-slaughter regimes, slaughterhouse surveillance, pre−/post-movement tests and movement restrictions, the incidence and geographic spread of bTB increased steadily in parts of England and Wales from the mid-1980s up to 2010, when they reached a plateau (Godfray et al., 2018). An overall declining trend in prevalence rates has been observed in England in the past 5 years, although this decrease has been heterogeneous (Gov.uk, 2024). Whilst many countries and regions have successfully eradicated or significantly reduced the disease using similar schemes (e.g., Scotland gained Official TB Free status in 2009), control efforts are more challenging in areas in which reservoirs of infection exist in wildlife (Palmer, 2013; Allen et al., 2018), as for instance in populations of the European badger (*Meles meles*) in GB (Godfray et al., 2013) and in possum in New Zealand (Livingstone et al., 2015). Furthermore, bTB poses a public health risk due to its zoonotic nature (Olea-Popelka et al., 2017; WHO, FAO, and WOAH, 2017), although the risk of human infection remains low in most developed countries since the introduction of milk pasteurisation (Müller et al., 2013).

Bovine TB causes a chronic respiratory disease with a pathology characterised by the development of granulomas in affected tissues of the infected animal (Domingo et al., 2014). *Mycobacterium bovis* (*M. bovis*), which causes bTB, is a member of the *Mycobacterium tuberculosis* complex (MTBC), a group of closely related and highly clonal organisms with over 99.9% identity in their genomes (Brosch et al., 2000). Other members of the MTBC include *Mycobacterium tuberculosis* and *M. africanum*, which are mostly human-adapted, and several animal-adapted species or ecotypes such as *M. microti, M. caprae, M. pinnipedii, M. mungi, M. orygis*, and *M. suricattae* (Fabre et al., 2004; Smith et al., 2006; Alexander et al., 2010; van Ingen et al., 2012; Parsons et al., 2013; Rodriguez-Campos et al., 2014). Although *M. bovis* has been associated primarily with cattle, it exhibits a wide range of wild mammalian hosts such as badgers, brush-tail possums, deer, elk, wild boar and water buffalo, as well as farmed or park deer, goats, pigs, sheep and South American camelids (Delahay et al., 2002; Fitzgerald and Kaneene, 2013).

Molecular typing methods have been extensively used in many countries to characterise and monitor the spread of *M. bovis* (Collins et al., 1986; Skuce et al., 2005; Tsao et al., 2014; Hauer et al., 2015). Earlier studies used traditional genetic markers to define four major clonal complexes carrying distinct deletion, spoligotype and single-nucleotide polymorphism (SNP) signatures. The clonal complexes African 1 (Af1) and African 2 (Af2) were discovered in West and East Africa, respectively (Müller et al., 2009; Berg et al., 2011), while European 2 was found to be predominant in the Iberian Peninsula and Brazil (Rodriguez-Campos et al., 2012). The European 1 (Eu1) clonal complex revealed a global distribution with prevalence in the United Kingdom (UK) and Ireland, and former trading countries of the UK (Smith et al., 2011).

In GB, spoligotyping, the most common typing technique of the MTBC, had been routinely used by the Animal and Plant Health Agency (APHA) for over 25 years as part of the bTB surveillance programme, complemented by multiple loci variable number tandem repeat (VNTR) analysis since 2005. Spoligotyping detects polymorphisms in the presence or absence of unique spacer sequences within the direct repeat (DR) region of the MTBC genome (Groenen et al., 1993) while VNTR measures variation in the number of tandem repeats across multiple repetitive regions of the genome (Frothingham and Meeker-O’Connell, 1998). The combined output of these two methods determines the genotype of an isolate, information that has supported epidemiological investigations at the individual incident level and allowed for large-scale analysis of the population structure and spread of bTB at regional and national levels (Waller et al., 2022).

Based on this type of data, it has been shown that the different genotypes found in Britain are geographically localised, leading to the hypothesis that the observed diversity of *M. bovis* is the result of a series of clonal expansions of specific genotypes following the substantial population reduction in the 1950s and 1960s due to the statutory eradication programme (Smith et al., 2003; Smith et al., 2006). The regional localisation of genotypes has been captured by the so-called home ranges; geographical areas where particular genotypes are commonly observed. In 2020, over 96% of isolates collected in Britain were represented by 27 genotypes, of which 53% were assigned to the three most frequent genotypes (GB genotype 25:a [which corresponds to a strain with a specific VNTR variant of spoligotype SB0129], GB genotype 17:a [as above but spoligotype SB0263] and GB genotype 11:a [as above but spoligotype SB0274]) displaying an extensive distribution in the South West and West of England and Wales (Gov.uk, 2021).

Although spoligotyping coupled with VNTR typing have proved to be invaluable in identifying groups of genetically related strains over larger geographical scales and in tracing the source of infection, particularly in cases in which long-distance cattle movement has been involved (Skuce et al., 2010), their discriminatory power is limited since they measure variation only at a small portion of the genome. This has been particularly problematic in areas of high endemicity, such as the high-risk area (HRA) of England, where certain genotypes are widespread, at the within-or between-herd level of transmission that involves much finer-scale genetic differences, as well as at multi-host species systems, in which finer resolution could help infer the direction of transmission. Moreover, due to their homoplastic nature, identical spoligotype or VNTR patterns can arise by convergent evolution rather than by evolutionary descent and therefore phylogenetic relationships cannot be reliably inferred (Warren et al., 2002). A new spoligotype pattern arises from the loss of one or more spacers in the DR region of a replicated bacterial genome, which due to the clonal nature of *M. bovis* is passed on to all its descendants, making it a suitable phylogenetic marker (Smith et al., 2003). However, because the frequency of these loss of spacer(s) mutation events is not as common as other mutations (e.g., SNPs), spoligotyping has limited discriminatory power. On the other hand, VNTR typing offers additional resolution since it involves multiple loci but is not as reliable phylogenetically since the number of repeats in the VNTR loci can increase or decrease, making it highly homoplastic.

Technological advances and continuously decreasing costs have made it possible for whole-genome sequencing (WGS) to be used as a diagnostic tool for outbreak investigations both in human TB (Gardy et al., 2011; Roetzer et al., 2013) as well as in bTB (Biek et al., 2012; Trewby et al., 2016; Price-Carter et al., 2018). WGS offers higher resolution compared to conventional typing methods as it assesses variation across the entire bacterial genome, making it possible to distinguish between isolates with identical genotypes. The greater degree of discrimination provided by WGS allows for more accurate reconstruction of risk pathways underlying transmission, especially when combined with high-quality epidemiological data, promising to offer a better understanding of the persistence and spread of bTB. For this reason, APHA implemented WGS in 2017 and officially replaced genotyping in 2021, following the recommendation from the review of the UK Government’s strategy to achieve officially bTB free status in England by 2038, that WGS should be used routinely as a surveillance tool (Godfray et al., 2018).

In this study, we analysed genome sequences from ~3,052 *M. bovis* isolates collected across GB to characterise their genomic diversity and population structure. Our phylogenetic reconstruction demonstrates that the *M. bovis* population in Britain is more diverse than previously thought and can be divided into seven major groups, with one of them subdivided further into 29 clades that differ from each other by at least 70 single-nucleotide polymorphisms (SNPs). Based on the obtained phylogenomic structure, we propose a SNP-based classification system to replace the traditional genotype scheme. We compare the two methods, discuss the benefits of WGS as well as provide a case study to showcase the insights that can be gained by higher resolution genome-wide data when combined with epidemiological information.

## 2 Materials and methods

### 2.1 Sampling and laboratory methods

As part of the bTB statutory surveillance programme, bTB culture-positive isolates collected during routine surveillance were characterised by spoligotyping since the late 1990s with VNTR-typing of six loci implemented in 2005. Whole-genome sequencing was implemented in 2017 in parallel with genotyping up until 2021, when WGS officially replaced genotyping. For our dataset, we selected sequences from 3,052 isolates collected between 2000 and 2023. The majority of sequences were routine surveillance isolates sampled across GB in 2022 (*n* = 2,615) (including bovines and non-bovines) capturing the diversity of the contemporaneous *M. bovis* population, while isolates pre-dating 2022 were added to ensure that most prevalent genotypes in GB were represented in our dataset.

Samples were inoculated on modified 7H11 slopes (Gallagher and Horwill, 1977) for 6–12 weeks and a single colony was harvested for molecular characterisation. These procedures were performed in Containment Level 3 (CL3) laboratories located at the Department of Bacteriology at APHA. Heat-inactivated isolates were spoligotyped and VNTR-typed following the methods described in Cadmus et al. (2011). Paired-end libraries were constructed directly from the heat-inactivated material without a DNA extraction or DNA purification step using the Nextera XT DNA Library Preparation Kit (Illumina, Cambridge UK) and sequencing was performed at the Central Unit for Sequencing and PCR (CUSP) at APHA on an Illumina MiSeq or NextSeq 500/550 instrument generating 150 bp paired-end reads. Isolates sampled prior to 2017 (*n* = 251), were first retrieved from the frozen bTB archives at APHA and re-cultured for up to 12 weeks, followed by sequencing of the heat-inactivated material as described above. Sequencing reads were deposited in the European Nucleotide Archive (ENA; accession number: PRJEB81540).

### 2.2 Sequencing data processing

Raw sequencing reads were processed by APHA’s in-house pipeline for *M. bovis* sequencing data, btb-seq^1^ with a few modifications as described below. Duplicate reads were removed by FastUniq V1.1 (Xu et al., 2012) and adaptors and low quality bases (using a sliding window of 10 bp and minimum average quality per base of 20) were removed using trimmomatic 0.38 (Bolger et al., 2014). The filtered reads were then mapped onto the updated version of the AF2122/97 reference genome (NC_002945.4; Malone et al., 2017) using BWA mem v0.7.17 (Li, 2013) with default settings. Samples with <90% of reads mapped to the reference genome indicate contamination from other organisms and were therefore not included in further analysis. SAMtools v1.10 and BCFtools 1.10.2 (Danecek et al., 2021) were used to perform variant calling and normalisation. The following filters were applied: minimum mapping quality of 30, minimum base quality of 10, minimum read depth at variant sites of 10, and minimum support for alternative or reference allele of 0.9. Regions that are problematic for SNP calling, such as repeat regions, IS elements, PPE and PE genes (Ates, 2020) as well as poorly covered regions were masked (positions listed in Supplementary Table 1). Poorly covered regions were determined by comparing 89 high-quality genome sequences and identifying regions of the genome that had insufficient read depth or insufficient support for the alternative or reference allele (Ns). An exponential decay function was applied to define the threshold of the density of Ns (number of genome sequences with an N at a given position) depending on the window size of Ns. This means that shorter N windows had a higher threshold (threshold of 0.08 for N window size = 1) while larger N windows had lower thresholds (threshold towards 0.04 for larger window sizes). Regions with Ns that exceeded these density thresholds based on their window size were masked. Moreover, SNPs that were found to be within 10 bp from each other were removed as they tend to be associated with repetitive regions (Gardy et al., 2011). The consensus genome sequences were combined in a multi-fasta alignment and snp-sites v2.5.1 (Page et al., 2016) was used to extract all polymorphic sites in our dataset.

### 2.3 Phylogenetic reconstruction

Maximum-likelihood (ML) phylogenies were constructed in IQ-Tree 2.2.0-beta (Minh et al., 2020) using the-fconst option to correct for ascertainment bias in our SNP-alignment. The K3Pu + F + R5 substitution model was used in our phylogenetic analyses, which was selected by IQ-Tree’s extended ModelFinder (Kalyaanamoorthy et al., 2017) as the third most optimal model. UNREST+ +FO + R5 and TVM + F + R5 were the top two selected models but due to their added complexity, they failed to converge in a reasonable amount of time. To verify the validity of internal nodes, a total of 1,000 ultrafast bootstrap replicates (Hoang et al., 2017) were performed. The sequencing data from a *Mycobacterium caprae* (*M. caprae*) isolate (accession number SRR7617662) was processed as described above and included in the phylogenetic analysis to root the tree. The ML phylogeny was annotated in TreeViewer v2.2.0 (Bianchini and Sánchez-Baracaldo, 2024).

Given the large number of sequences in our alignment, Treemmer v0.3 (Menardo et al., 2018) was used to remove redundant branches and better visualise the phylogenetic tree without compromising its genetic diversity. To achieve this, the maximum number of samples per WGS clade (as defined below – see section “Results and Discussion”) was limited to 50. This was done to downsize WGS clades with a large number of genetically highly similar sequences while maintaining the number of sequences in underrepresented WGS clades. The phylogenetic tree of the trimmed dataset was constructed in IQ-Tree as described above.

### 2.4 Clade-specific SNPs

Unique SNPs were identified that were exclusively shared by all isolates in a defined WGS clade but not present (i.e., they were not polymorphic) in any of the other clades. For this analysis, high-quality sequences per clade were selected (> 99% of reads aligned to the reference genome with >50-fold depth of coverage and > 99% of the reference genome covered; with a few exceptions for some of the clades; see below). The ancestral state reconstruction feature in Mesquite v3.81 (Maddison and Maddison, 2023) was employed to infer the sequence of the most recent common ancestor of each WGS clade, which were then used to detect unique SNPs per clade. To facilitate this analysis (Mesquite cannot handle very high numbers of sequences), we selected 10 high-quality sequences per clade from the original (i.e., non-trimmed) ML phylogeny (listed in Supplementary Table 2). For clades that showed higher diversity comprising relatively deeply diverged sub-clades (e.g., B5-11), all high-quality sequences within those clades were included. For clades that were underrepresented, all available sequences within those clades were included irrespective of the quality criteria above (B6-12, *n* = 10; B6-21, *n* = 8; B6-22, *n* = 10; B6-23, *n* = 9; B6-31, *n* = 10; B6-41, *n* = 10; B6-51, *n* = 10; B6-53, *n* = 10; B6-61, *n* = 2; B6-81, *n* = 3; B6-88, *n* = 4; B6-92, *n* = 9; B7-11, *n* = 3). These sequences still had an average genomic read depth of at least 23-fold, over 91% of the reads aligned to the reference genome, and over 99% of the genome covered. All polymorphic positions from this alignment (*n* = 4,803) were used to generate a maximum-parsimony (MP) phylogeny in MEGA v11.0.11 (Tamura et al., 2021) and the nodes representing the most recent common ancestor of each of the defined clades were selected on the output tree file, which was used as the input for Mesquite. The sequence of the selected ancestral nodes were inferred by Mesquite v3.81 under the parsimony model. Polymorphisms that were unique in each of the ancestral sequences were extracted using a presence/absence matrix, representing unique SNPs in each of the defined clades. The list of clade-specific SNPs was tested against the entire dataset and the clade of each sequence determined from scanning these sites on the WGS data was compared to the phylogenetic clade they grouped within, in the original ML tree (Supplementary Figure 1).

### 2.5 *In silico* spoligotyping

*In silico* spoligotyping was performed on the raw sequencing reads using SpoTyping v2.0 (Xia et al., 2016) and the output was converted to the respective SB number in the *M. bovis* spoligotype database.^2^ Spoligotype patterns that had not been observed before were given a new unique SB number upon request from the database above. The genotypes of isolates that had been spoligotyped and VNTR typed in the laboratory were retrieved from APHA’s cattle testing SAM database.

### 2.6 Pairwise SNP distances

Between-and within-clade pairwise SNP distances were estimated using snp-dists^3^ v 0.8.2. For the within-clade estimates, all pairs of isolates within a clade were compared. For the between-clade estimates, all sequences from one clade were compared against all sequences from the clade that appeared to be the closest neighbour in the original ML phylogeny (e.g., B1-11 against B3-11 and B6-81 against B6-82). Between-and within-clade pairwise genetic distance distributions were visualised with pyplot in the matplotlib module (Hunter, 2007).

### 2.7 Clade-specific SNPs functional analysis

To investigate the potential functional effect of the clade-specific SNPs, SNPeff v. 5.2c (Cingolani et al., 2012) was used with *M. bovis* AF2122/97 (gca_000195835) as the annotated reference database. This tool outputs variant and genetic information for all variants against the reference genome and predicts the effect of variants found on coding regions. Additional information about the genes on which variants with a moderate (non-disrupting) or high (disrupting) predicted impact were found, such as associated biological process, molecular function and cellular component, were obtained from Uniprot (Consortium, T.U, 2022), using annotation information from *M. bovis* in the first instance and prioritising reviewed entries. For genes where annotation information did not exist for *M. bovis*, the search was expanded to *M. tuberculosis* and then to all mycobacteria.

### 2.8 Case study

Our dataset included WGS data from three isolates collected from a farm in the southwest (SW) of England that had an outbreak in 2022. This data was analysed together with isolates in our database that were found to be within the same WGS clade as the three isolates and a MP tree was generated in MEGA v11.0.11 (Tamura et al., 2021). The phylogenetic tree was interrogated to identify closely related isolates and integrated with cattle location and testing data, stored in APHA’s SAM database, as well as cattle movement data from the Department of Environment, Food and Rural Affairs’ (DEFRA)‘s cattle tracing system (CTS), to determine the origin of infection and potential transmission routes in this incident.

## 3 Results and discussion

### 3.1 Assembling and processing the dataset

In this study, genome sequences from a total of 3,052 *M. bovis* isolates from across GB were analysed covering a time span from 2000 to 2023. A total of 2,615 isolates (85.7%) were sampled and sequenced in 2022 while 344 isolates (11.3%) were also traditionally spoligotyped and VNTR-typed as part of the routine surveillance scheme up to 2021. This dataset was selected to obtain a representative sample of the *M. bovis* population circulating in GB, including representatives of the most frequently observed genotypes (Gov.uk, 2021). The vast majority (*n* = 2,889; 94.7%) of isolates originated from cattle sourced from a total of 1,923 herds while other host-species, including badgers, deer, alpacas, pig, sheep and cats, were represented in our dataset by 150 samples in total (*n* = 13 had unknown host-species). Single samples per animal were analysed.

**Figure 1:**
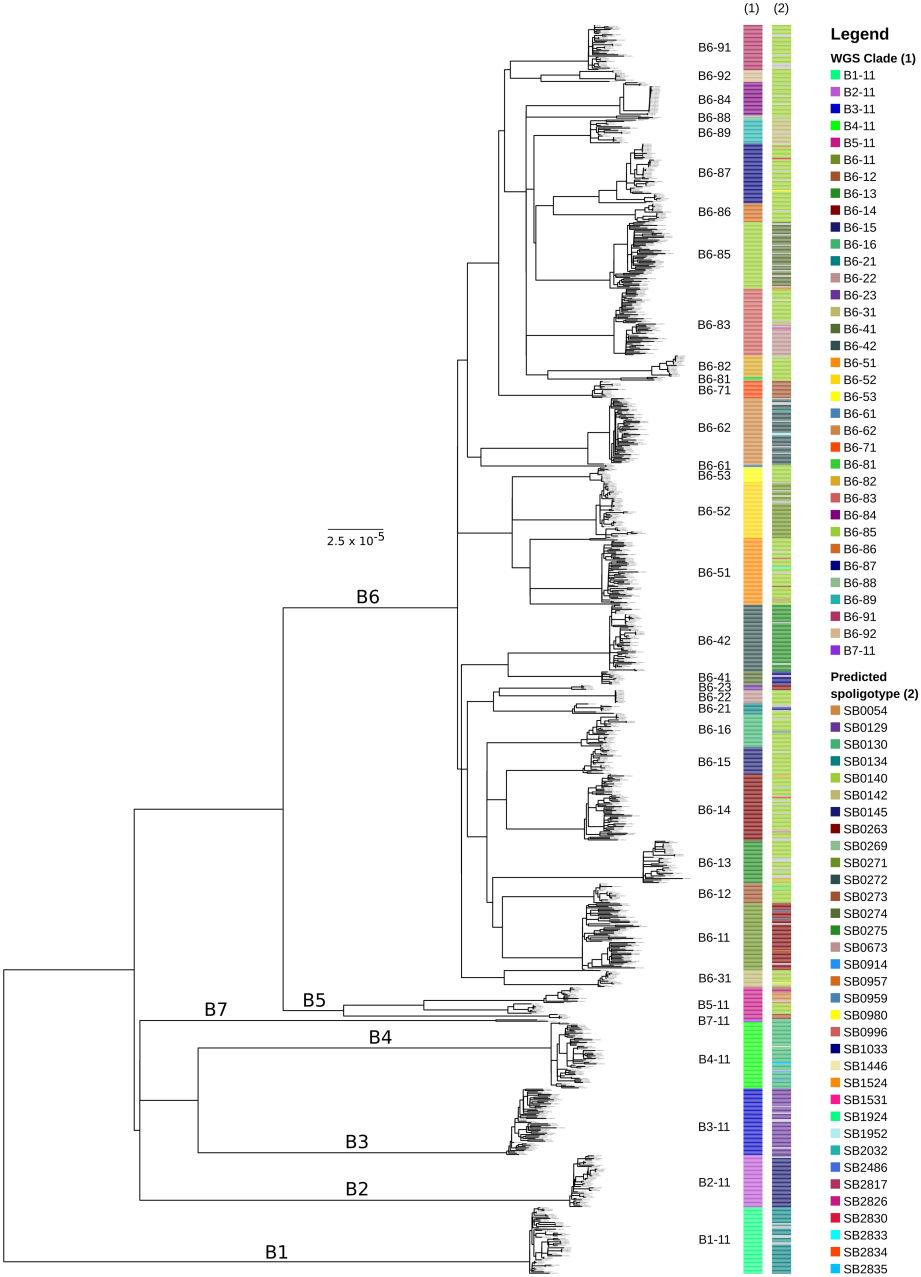
Maximum-likelihood phylogeny of trimmed *Mycobacterium bovis* dataset (*n* = 938 sequences) from across Great Britain. The outgroup of the tree (*M. caprae* sequence SRR7617662) has been removed for better visualisation. The scale bar represents units of substitutions per site. Major clades (B1–B7) are labelled on the tree and minor clades (B6-11–B6-92) are shown next to the tips of the tree. WGS clade and *in silico* predicted spoligotype for each isolate are indicated in the bars next to the phylogeny (from left to right). Grey bars on the *in silico* predicted spoligotype annotations mean that a unique spoligotype pattern was identified that had not been observed for any other isolate (singletons). Bootstrap support values for major internal nodes including nodes that represent the most recent common ancestor of each of the WGS clades are over 95%.

Samples with <90% of reads mapped to the reference genome (*n* = 128; 4.2%) were excluded from further analysis. The remaining dataset (*n* = 2,924) had an average depth of coverage of 107-fold (range: 13–578) with >99% of the genome covered (average = 99.8%). Sequences that had no calls (Ns) in more than 50 polymorphic positions were removed, resulting in a total of 2,823 sequences in our alignment, which was used to reconstruct a ML phylogeny (Supplementary Figure 1). Figure 1 shows the reduced ML phylogeny (938 sequences; 8,834 SNPs) generated by Treemmer that removes redundancy in our dataset, allowing for better visualisation.

### 3.2 Phylogenetic structure and estimates of genomic diversity

**Figure 2:**
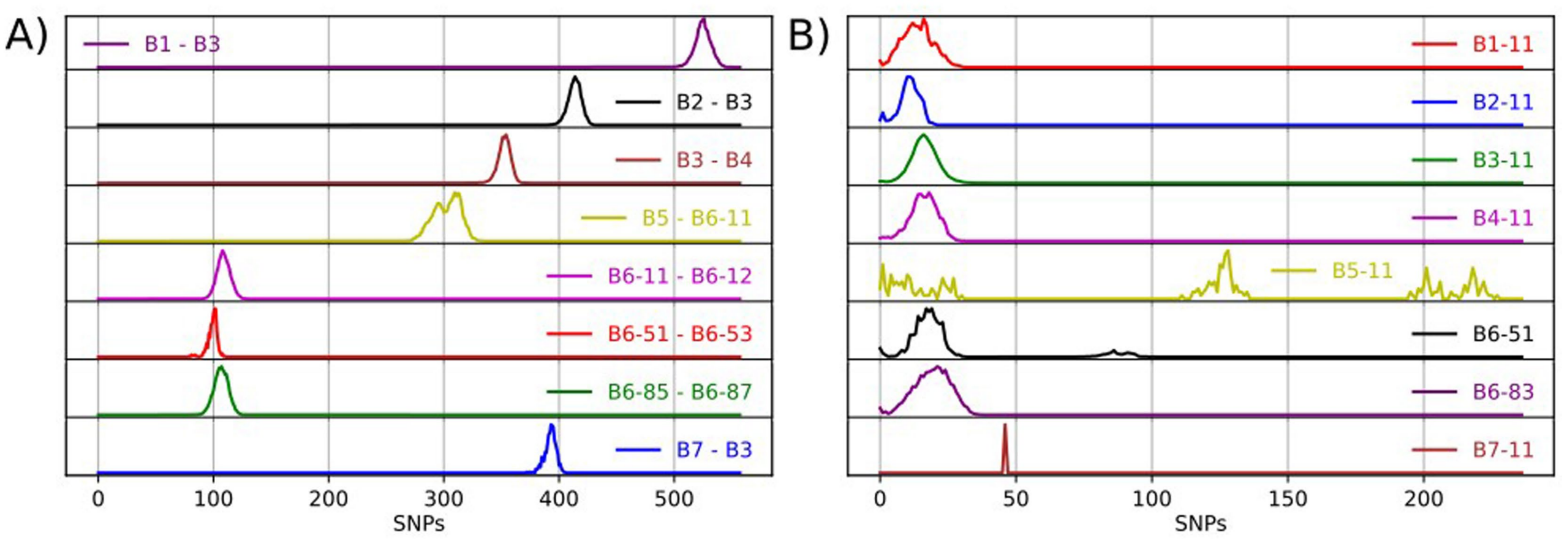
Frequency distribution of pairwise SNP distances between isolates from select WGS clades **(A)** and between isolates within a WGS clade **(B)**.

Seven major clades were identified, named B1 – B7, which are monophyletic and have diverged considerably from each other (Figure 1; Supplementary Figure 1). Major clades B1 - B5 and B7 represent deeply diverged lineages with pairwise SNP distances of at least 350 SNPs between them (Figure 2A; Supplementary Figure 2). B1 – B4 and B7 exhibit limited diversity, while B5 appears to be more diverse and can be split further as and when additional data becomes available (Figure 2B; Supplementary Figure 3). Major clade B6 appears to be highly diverse and was further subdivided into 29 minor monophyletic clades (B6-11 to B6-92; Figure 1) that are well-supported (bootstrap support values >95%) and separated by at least 70 SNPs (Figure 2A; Supplementary Figure 2). These clades were defined visually and were originally guided by our previous knowledge of the genotypes found in Britain. However, as inconsistencies between the phylogenomic and genotyping data were observed (discussed in section 3.3 below), we deviated from using the genotypes and applied a SNP distance threshold of 70 SNPs, which was the observed minimum distance between well-supported minor clades. The nomenclature presented above uses simple alphanumeric coding, where “B” stands for bovis, the first digit denotes the major clade and the next two digits denote the minor clade (groups within the major clade). This system is hierarchical, indicating relatedness between clades (e.g., B6-11 and B6-12 are more closely related to each other than to B6-81 and all three are more closely related to each other than to B4-11), and can be expanded to allow for potentially newly identified clades to be included and existing clades to be further subdivided.

The topology of the tree with most clades appearing “flat” and separated by deep branches suggests that they have gone through recent population expansions. Although we did not attempt to date these expansions, this observation is consistent with the theory of a series of clonal expansions in GB following the severe reduction in the population size of *M. bovis* in the 1950s and 1960s caused by the cattle testing and eradication programme (Smith et al., 2003; Smith et al., 2006). In future work, we aim to retrospectively sequence historical isolates from APHA’s bTB collection going back to the late 1980s to enhance the temporal signal in our dataset, which will allow us to generate dated phylogenies and estimate the timings of expansions and divergence of the defined WGS clades.

**Figure 3:**
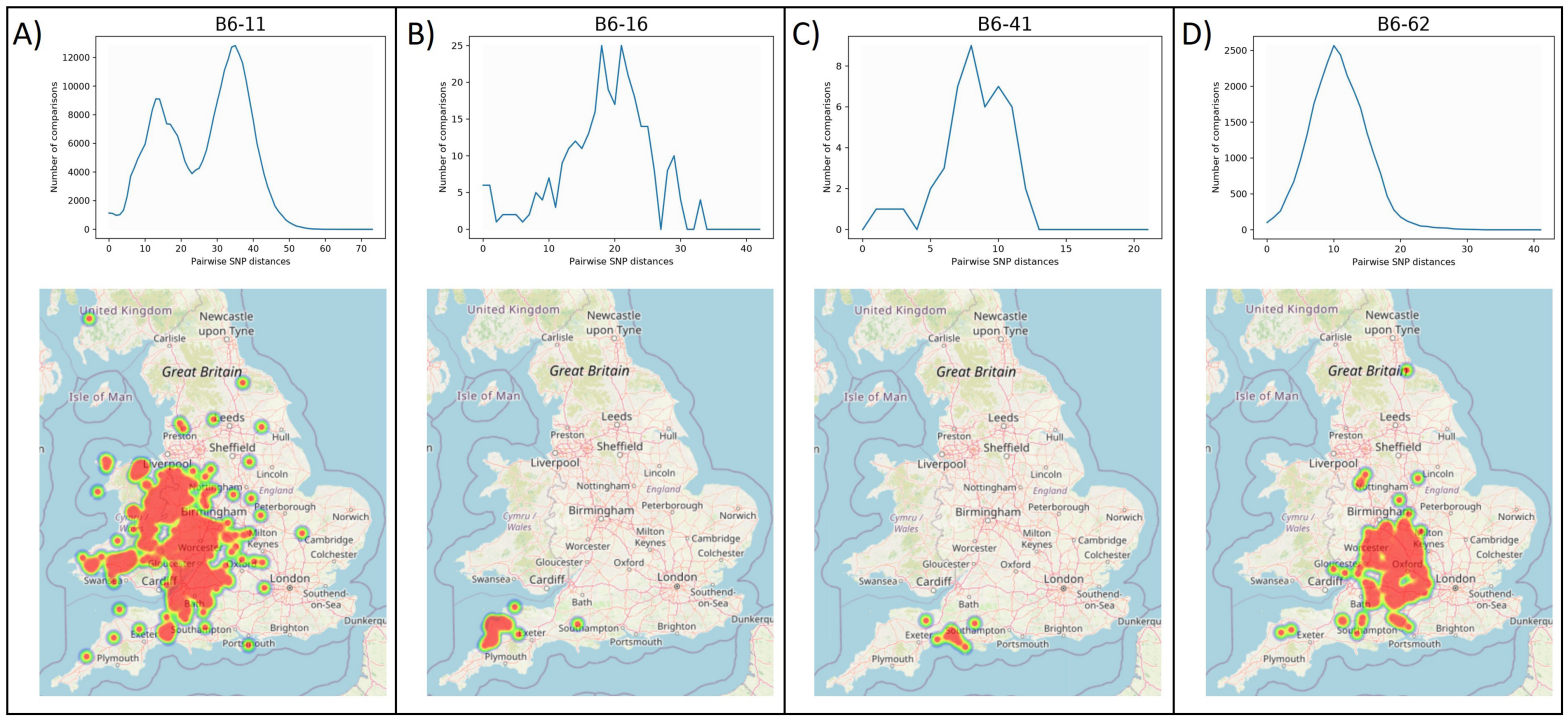
Frequency distributions of within-clade pairwise SNP distances (top) and geographical ranges (bottom) for clades B6-11 **(A)**, B6-16 **(B)**, B6-62 **(C)** and B6-41 **(D)**. B6-11 and B6-62 **(A, D)** represent widespread clades with relatively high and low levels of genetic diversity, respectively, while B6-16 and B6-41 **(B, C)** represent clades with constrained geographical ranges and medium or low diversity, respectively. The y-axis in the frequency distribution plots shows the number of pairwise comparisons and the x-axis shows pairwise genetic distances in number of SNPs. Note that the scale of the x-axis differs in each of the clades.

The geographic distribution of each WGS clade was examined, by plotting the location of the source holdings of the cattle isolates using the online tool grid reference finder^4^. Similar to the geographic localisation previously observed for the different genotypes in GB (Smith et al., 2003), which led to the definition of home ranges, i.e., areas in which certain genotypes are expected, most WGS clades appear to have a defined geographical distribution. Geographical range does not appear to correlate with genomic diversity; clades with a limited geographical range (e.g., B6-16 and B6-41 in Figure 3) exhibit varying levels of genetic diversity. B6-41 exhibits low levels of diversity (pairwise SNP distances <15 SNPs) while B6-16 exhibits higher diversity (average: 18 SNPs and max: 35 SNPs). Clades that are geographically more widespread occupying large parts of the HRA, also exhibit varying levels of genomic diversity. For instance, B6-11, the most prevalent and widespread clade, displays high genomic diversity (up to 65 SNPs). On the other hand, B6-62, another clade with extensive geographic distribution covering parts of 12 counties, displays lower diversity (average: 11 SNPs and max: 32 SNPs), which is on average lower than that observed in B6-16. This needs to be further explored statistically in future studies, testing for isolation by distance, as well as comparing the phylogenomic data with cattle movement direction and frequency data (either specific data for the source holdings or typical patterns for farm types/locations) to better understand their role in geographical spread.

A few of the WGS clades have bi-or multi-modal distributions of genetic diversity with one or more of the observed peaks within the expected range of within-clade pairwise SNP distances (0–65 SNPs) and additional peaks at a higher range (Supplementary Figure 3; B5-11, B6-31, B6-51, B6-92). The latter peaks are mostly due to the presence of diverged isolates within these clades, which potentially represent under-sampled *M. bovis* variation within our dataset, most likely originating from imported infection. In fact, a few of our clades (e.g., B5-11, B6-89, B7-11) are associated with imported infected animals as evidenced from movement history and could therefore be considered as non-endemic. Despite their large genetic distances, these isolates were not separated out from their respective WGS clades since their numbers were limited. However, expanding our sampling spatially, including from countries where imported animals are likely sourced, as well as temporally could possibly fill in those gaps and allow us to further split some of these WGS clades into distinct groups. The nomenclature presented above uses simple alphanumeric coding that can be expanded to allow for potentially newly discovered clades to be included and existing clades to be further subdivided.

### 3.3 Correlation of WGS clades and spoligotypes

Estimates of diversity based on the genome-wide data were compared against estimates based on *in silico* predicted spoligotyping. A detailed comparison between lab-based and *in silico* predicted spoligotypes is provided in the Supplementary Information. Overall, there appears to be agreement between the defined WGS clades and previously established spoligotypes. This is shown in Figure 1 as well as in Table 1 that maps the spoligotypes observed in each WGS clade. However, almost half of the WGS clades include more than one spoligotype with the majority of isolates coming from a single spoligotype (e.g., SB0140 [GB type 9] in clade B6-87) and a small proportion of isolates from different spoligotypes (SB0054 [GB type 65], SB0980 [GB type 103], and SB2830 in clade B6-87). By examining these spoligotype patterns, we can see that single mutation events in the most prevalent spoligotype within a clade have given rise to the less common spoligotypes that are present within the same clade via the loss of a single or multiple contiguous unique spacers within the DR region (see Supplementary Figure 4 for example above), demonstrating that isolates with different spoligotypes can in fact be genetically closely related to each other, contrary to what their different spoligotypes might have suggested.

**Table 1:** WGS clades and their corresponding spoligotypes based on the lab-inferred and in silico predicted spoligotypes of the isolates in our dataset.

B1-11	SB0134								
B2-11	SB0145								
B3-11	SB0129	*SB1551*							
B4-11	SB0130	*SB1446*	*SB0914*	*SB2835*					
B5-11	SB0273	SB0054	SB0140	*SB2817*					
B6-11	SB0263	*SB0959*	*SB0957*	*SB1415*	*SB2059*	*SB2728*	*SB0661*	*SB0977*	*SB2032*
	*SB2828*	*SB2829*							
B6-12	SB0140	*SB1924*							
B6-13	SB0140	*SB1524*							
B6-14	SB0140	*SB0996*	*SB0139*	*SB1531*	*SB0054*	*SB0980*	*SB1924*	*SB0269*	*SB1524*
	*SB0673*	*SB2830*	*SB2826*						
B6-15	SB0140								
B6-16	SB0140	*SB2486*							
B6-21	SB0140	*SB1033*							
B6-22	SB0140								
B6-23	SB0263								
B6-31	SB0140	*SB0142*	*SB0980*						
B6-41	SB1033								
B6-42	SB0275								
B6-51	SB0140	*SB0273*	*SB0996*	*SB0269*	*SB2833*				
B6-52	SB0271								
B6-53	SB0140								
B6-61	SB0140								
B6-62	SB0272	*SB2032*	*SB2831*	*SB1952*	*SB2833*				
B6-71	SB0273								
B6-81	SB0140								
B6-82	SB0140								
B6-83	SB0673	SB0140	*SB0269*	*SB2486*	*SB2826*				
B6-84	SB0140	*SB0269*							
B6-85	SB0274	*SB1499*	*SB2827*	*SB2832*	*SB2834*				
B6-86	SB0140								
B6-87	SB0140	*SB0054*	*SB0980*	*SB2830*					
B6-88	SB0140								
B6-89	SB0142								
B6-91	SB0140								
B6-92	SB0140								
B7-11	SB0130								

*Italic* font indicates less frequent spoligotypes while non-italic font indicates prevalent spoligotypes within each of the WGS clades. Spoligotypes that were observed only once (singletons) are not shown in this table.

There are also spoligotypes/genotypes that are polyphyletic and therefore split into multiple WGS clades across the phylogeny. For instance, SB0273 [GB type 13] is observed in clades B5-11, B6-51 and B6-71, which do not form a monophyletic group (Figure 1; Supplementary Figure 1 and Supplementary Table 1). Other spoligotypes observed in polyphyletic groups are SB0140 [GB type 9], SB0130 [GB type 21], and SB0263 [GB type 17]. This can be explained by a process by which an ancestral lineage with a prevalent spoligotype pattern gave rise to a large number of lineages (i.e., WGS clades), some of which share the same spoligotype pattern (i.e., no loss of spacer(s) events in their DR region during their evolution) while others evolved different spoligotype patterns due to the loss of a single or multiple spacers in their DR region. This process can also lead to homoplastic mutations in the direct repeat region, by which identical spoligotypes can emerge in phylogenetically unrelated lineages (Smith et al., 2006). Spoligotype SB0140 (GB type 9) is noteworthy; it is historically the most prevalent spoligotype in GB and is believed to have given rise to other common spoligotypes as well as diversified into many lineages (Smith et al., 2006). In our analysis, it is observed in 21 different WGS clades (Table 1; Figure 1; Supplementary Figure 1) across the phylogeny demonstrating that isolates of this type can be genetically quite different from one another despite their identical spoligotype. Interestingly, the majority of the home ranges of the WGS clades in which spoligotype SB0140 (GB type 9) is present, appear to be mostly confined and isolated from each other (Gov.uk, 2022), further supporting the evidence from the WGS data that these clades are genetically distinct.

These findings highlight that some of the assumptions based on conventional genotyping that were made in the past to inform epidemiological analyses might have been incorrect with potentially negative impact on case management, resulting in less effective control measures. Similar inconsistencies between WGS lineages and spoligotypes or genotypes have been reported previously (van Tonder et al., 2021) and show that WGS not only provides higher resolution, but also more accurate and reliable inference of genetic relatedness compared to conventional typing methods.

### 3.4 SNP markers for the *M. bovis* population in GB

Based on the WGS clades defined in Figure 1, a set of 2,058 polymorphic positions that are unique in each of the 35 clades, were identified from a subset of high-quality sequences (*n* = 358). Due to the strict clonality of the MTBC (Smith et al., 2006), these SNPs can be used as markers to assign isolates to the 35 different WGS clades that capture the genetic diversity of *M. bovis* in GB (Supplementary Table 3). The number of specific SNPs per clade varies, with B6-51 exhibiting the lowest number of unique SNPs (*n* = 9) and B1-11 the maximum number of unique SNPs (*n* = 294). When this set of SNPs was tested against the entire dataset, 7 SNPs that were originally found to be unique in clade B6-85, were found to be specific for a subgroup within this clade and not for the entire clade. This was due to the subsampling that was performed for this analysis and the isolates that were selected to represent clade B6-85, which did not capture its entire diversity. Similarly, two SNPs that were each originally found to be unique in clades B1-11 and B6-85 were found to have mutated back to the reference call in a subgroup of isolates and a single isolate within each clade, respectively, which was again due to the subsampling performed for this analysis. These sites were therefore removed from the SNP set since they were polymorphic and not fixed in the clades above. For the remaining set of SNPs (*n* = 2,049 sites), when tested against the entire dataset (*n* = 2,823 sequences), SNP-based clade assignment was consistent with the phylogenetic clade in which each sequence belonged with an average of 99.1% matching ratio across each clade’s defining SNPs (range: 54–100%). Matching ratios lower than 100% were due to Ns in clade-defining SNP positions.

SNP-based tools or sets of lineage-specific SNPs have been published before to identify different members of the MTBC (Lipworth et al., 2019), to differentiate *M. bovis* from *M. caprae* and *M. orygis* (Zwyer et al., 2021), and to distinguish global lineages of *M. bovis* (Zimpel et al., 2020). Although these SNP sets are useful for characterisation of MTBC strains or *M. bovis* strains in a wider context, this present study identified unique SNPs that specifically characterise the diversity of *M. bovis* in GB. These SNP sets can further be used and expanded to include *M. bovis* strains isolated from neighbouring countries, as well as from other countries that may share similar genetic lineages due to cattle trade with the UK (Smith et al., 2011).

### 3.5 Annotation of the clade-specific SNPs

Annotation of the unique SNPs that define the 35 WGS clades identified in this study showed that 89% of the variants (*n* = 1,824) were located on protein-coding regions, of which 32.5% (*n* = 665) were synonymous and therefore assumed to be neutral. In addition, 12.2% (*n* = 250) were in intergenic regions and therefore non-coding but may have unknown regulatory functions. The remaining mutations were non-synonymous, meaning that the encoded amino acid is altered, a start or stop codon is lost, or a premature stop codon is introduced.

We focused on the mutations that were predicted to have high or moderate impact on the encoded protein by SNPeff (*n* = 1,133) and the genes they affect (Supplementary Table 3). High impact variants are assumed to be disruptive, causing protein truncation, loss of function or triggering nonsense mediated decay, while moderate impact variants are assumed to be non-disruptive, potentially changing the effectiveness of the protein (Cingolani et al., 2012). The highest number of such variants were found in genes that are involved in DNA synthesis and translation processes, as well as phosphorylation that plays an important role in the pathogen and host physiology, such as virulence, signalling and immune response (Wang and Xie, 2022). Other predicted genes that appear to be overrepresented among high and moderate impact variants are associated with processes such as cell wall organisation, the biosynthesis and transport of the cell wall lipid PDIM (phthiocerol dimycocerosates), secondary metabolite biosynthetic processes, and fatty acid and lipid biosynthetic processes. The latter have been shown to play an essential role in the formation of cell wall components, which are thought to be important in host-pathogen interactions, virulence and pathogenicity (Kinsella et al., 2003; Gago et al., 2018; Mondino et al., 2020). The cell wall lipid PDIM of *M. tuberculosis* has been shown to inhibit autophagy in mice, contributing to its virulence and immune evasion (Mittal et al., 2024).

Notably, 180 genes (of which 90 are of known function and 90 are of unknown function) were found to harbour more than one of these high or moderate impact mutations, largely defining different clades. One of these genes, which has eight clade-specific mutations (each specifying a different clade) is *whiA*, a gene encoding a DNA-binding protein that is involved in cell division and chromosome segregation in most Gram-positive bacteria. This gene has been shown to be essential for sporulation in *Streptomyces coelicolor* A3 (Aínsa et al., 2000) and to affect fatty acid composition of the membrane in *Bacillus subtilis* (Suarez, 2018). Another gene containing six clade-specific mutation is *pks12*, which has been predicted to encode a novel polyketide that is presented to CD1c-mediated T cells in *M. tuberculosis* and *M. bovis* Bacille-Calmette-Guerin and is therefore necessary for antigen production (Matsunaga et al., 2004). This gene has also been shown to be involved in PDIM production (mentioned above) and pathogenesis (Sirakova et al., 2003). Other genes containing four mutations are the following: *fhaA*, which plays a role in peptidoglycan biosynthesis and maintenance of the cell envelope integrity in mycobacteria (Viswanathan et al., 2017); *rsmH*, a member of the 16S RNA methyltransferase family, in which mutations are known to confer aminoglycoside resistance in other bacteria (Ledger et al., 2020); and *metY* that is involved in the biosynthesis of homocysteine, a sulphur metabolic pathway (trans-sulfuration) which is essential for survival and the expression of virulence in *M. tuberculosis* (Wheeler et al., 2008; Paritala and Carroll, 2013).

The observation that many of these genes are associated with virulence and pathogenicity in *M. bovis, M. tuberculosis* and/or other bacteria raises the hypothesis that the WGS clades identified in this study may exhibit phenotypic variation, which might have led to different evolutionary trajectories. However, experimental *in vitro* studies would be required to examine the functional effect of the clade-specific mutations as the above are merely computational predictions. Furthermore, genome-wide association studies and selection scans can in future be performed to investigate whether these mutations were driven to fixation owing to selection or random genetic drift.

### 3.6 Discriminatory power of WGS at the local scale

WGS data from a recent outbreak in 2022 on a farm (referred to as farm “A” from here on) in the HRA of England (SW) that had no history of TB incidents in the previous 10 years were analysed and integrated with location, cattle movement and testing data, in an attempt to determine the source of infection and risk pathway involved. Of the 10 animals that tested positive at the disclosing Single Intradermal Comparative Cervical skin test, three samples were collected for mycobacterial culture and sequencing, and subsequently three *M. bovis* isolates were all found to belong to clade B6-51. Based on the home range for clade B6-51, the incident was located within its expected geographic range, and the same clade had been identified in earlier outbreaks on nearby farms. Notably, badger culling had recently started in that area and a sick badger had been found on farm “A” a few months prior to the outbreak, leading to speculation that local spread via wildlife was a likely source attribution.

**Figure 4:**
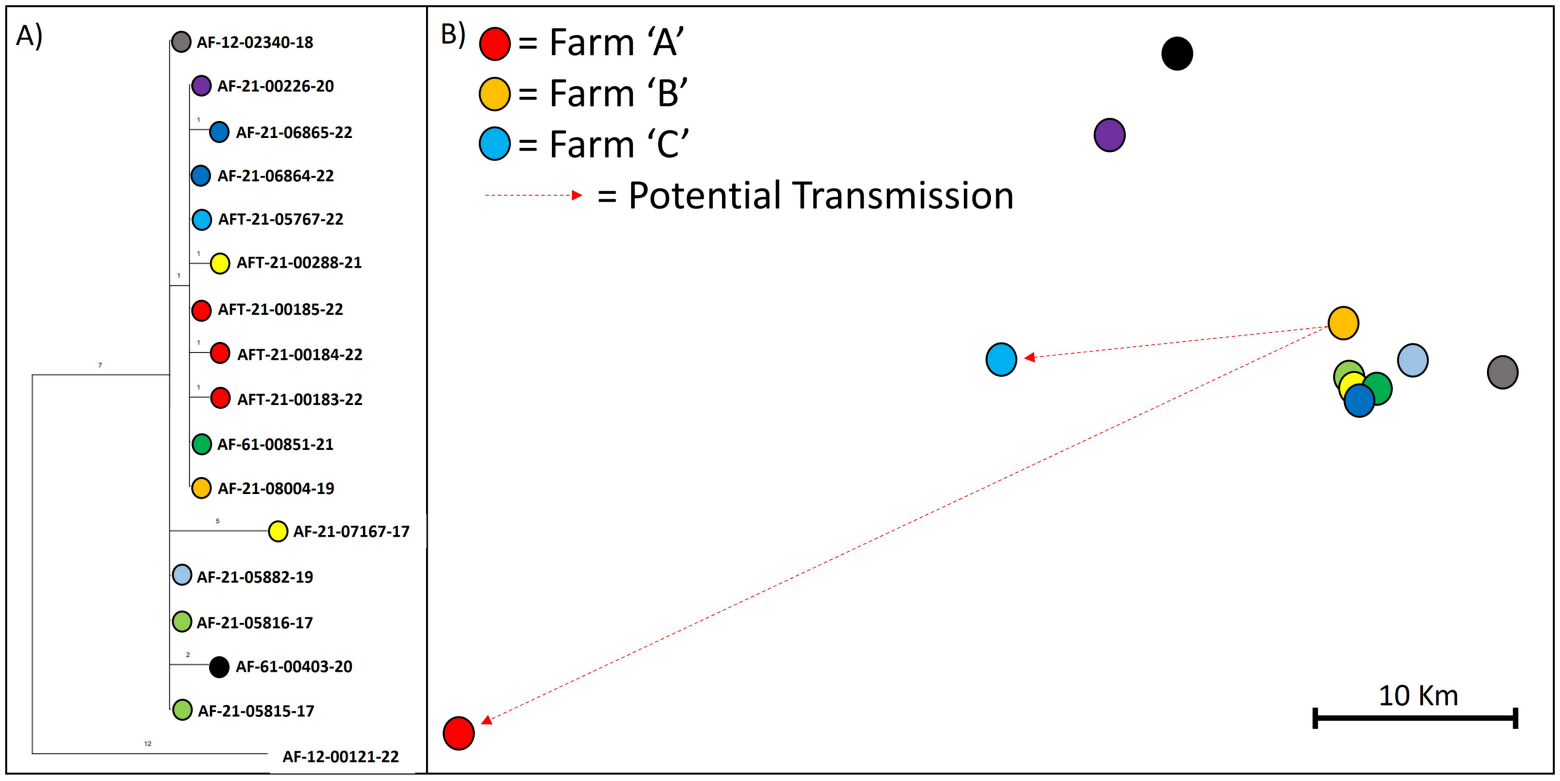
Maximum parsimony tree **(A)** and map **(B)** showing the phylogenetic relationships and relative location of the three isolates collected from a recent outbreak (farm A) in the SW of England. The tree **(A)** is a snippet from the phylogeny of WGS clade B6-51. Branch length indicates number of SNPs. Relative locations are shown on the map **(B)** to comply with data protection law in the UK.

Using the genome sequences of the three isolates from farm “A” and all other sequences within our dataset that were assigned to clade B6-51, we built a MP tree to reconstruct their phylogenetic relationships. Figure 4A shows that the three isolates differed by 1–2 SNPs from each other. Isolate AF-21-000185-22 was identical to five other isolates from incidents dating from 2019 to 2022, which were geographically clustered to the north-west of farm “A” at ~35 miles distance. The remaining two isolates were each one SNP away from the identical isolates with which they share a common ancestor (Figure 4B).

This phylogenetic analysis instigated the inspection of movements of the animals from which the identical isolates were collected; two animals were found to have originated from the same farm (referred to as farm “B” from here on) and overlapped in time for approximately 2 years and 7 months. The animal from which the 2019 isolate (AF-21-08004-19) was collected, was born on farm “B” in 2017 and lived there until December 2019, when it tested positive and was sent for slaughter. The animal from which the 2022 isolate (AF-21-05767-22) was collected, was also born on farm “B” in 2012 and lived there for 8 years and 9 months before it was purchased by another farm (farm “C”) via a local market (in September 2021). It was then disclosed as a reactor animal in April 2022 at a 12-month test on the purchase farm, following the conclusion of a previous outbreak on that farm, despite its clear pre-movement test on farm “B” in August 2021. It should be noted that this animal had also tested negative through 3 previous whole-herd short interval tests and a 6-month check test on farm “B”, after the disclosure of the 2019 isolate and two subsequent positive slaughterhouse cases.

Interestingly, by looking at the movement and testing data from farm “A,” it was discovered that farm “A” had purchased two animals from farm “B” in August 2021, which both had a positive skin test following their movement, in August 2022 and November 2022 respectively, but for which no bacterial genetic data is available. The animal disclosed in August 2022 did not undergo post-mortem examination as it was within its drug withdrawal period and slaughtered on farm. However, according to its clinical history, it had been losing weight and was dyspnoeic as well as unresponsive to antibiotics. The second animal disclosed in November 2022, did not exhibit visible lesions and was not sampled. Both these animals were born on farm “B” in September 2019 and March 2018, respectively, and had skin-tested negative 7 and 8 times, respectively, on that farm prior to their movement. Hence, the genomic and epidemiological data analysed together suggest that one or both of these animals were infected on farm “B”, and that at least one was an open case and source for other cattle on farm “A” upon their arrival.

By harnessing the higher resolution provided by WGS data and integrating it with epidemiological information, we were able to attribute the 2022 outbreak on farm “A” to cattle purchases in 2021 from “farm B.” Our findings show that this farm had not cleared infection after its own outbreak starting in 2019, despite three rounds of subsequent whole-herd short-interval testing and a 6-month check test. Identifying farm “B” as the source of infection would not have been possible by only looking at the gross-level WGS clade characterisation of these isolates or at the previously used genotypes; the isolates would have been considered to be within home range and local spread via wildlife would have been assumed as the most likely source of infection, given the recent sighting of a sick badger on farm. In contrast, our findings based on the genomic and epidemiological data indicate that infected animals missed during testing along with cattle purchases, led to this outbreak on a farm with no TB history in the previous 10 years, which has implications for control policy measures. It should be noted that this is not an isolated case, our investigations have shown many examples where within-home range transmission can be explained by local cattle movement, adding to the growing evidence that within-species transmission is considerably more prevalent than between-species transmission (Crispell et al., 2019; van Tonder et al., 2021; Rossi et al., 2022; Akhmetova et al., 2023; Canini et al., 2023). Our analysis further demonstrates that genomic similarity can be used to highlight potential transmission links, narrowing down epidemiological investigations, but also in the absence of other information, which is one of the major benefits of WGS.

## 4 Conclusion

This study characterised the genomic diversity of *M. bovis* across GB from the analysis of ~3,000 genome sequences. A total of 35 WGS clades was identified, based on which, a SNP-based classification system was designed that differentiates bTB isolates from GB more accurately and with higher granularity compared to previously used typing schemes. The genes harbouring these mutations and the processes they are associated with were discussed with potential implications for functional differences between the identified clades. The utility and high resolution of WGS at the local scale was demonstrated by analysing genome sequences from an outbreak in an endemic area of England, where source attribution remained challenging due to the limited discriminatory power of traditional typing methods. It is shown that WGS can be a powerful and informative tool when used alongside epidemiological data that can better determine transmission pathways, leading to more efficient case management policies and better-targeted control measures and interventions in the fight against bTB.

## Data Availability

The original contributions presented in the study are publicly available. This data can be found here: https://www.ebi.ac.uk/ena/browser/view/PRJEB81540.

## Footnotes

^1^ https://github.com/APHA-CSU/btb-seq

^2^ https://www.mbovis.org/database.php

^3^ https://github.com/tseemann/snp-dists

^4^ https://gridreferencefinder.com/
