# Supplementary material for: Enhanced analysis of the genomic diversity of Mycobacterium bovis in Great Britain to aid control of bovine tuberculosis
Source: Front Microbiol. 2025 Mar 25;16:1515906. doi: 10.3389/fmicb.2025.1515906 (PMC11975571; doi:10.3389/fmicb.2025.1515906)
Supplement: Supplementary file 1 [file Data_Sheet_1.docx]

Supplementary Material

# Supplementary Information

Lab-based spoligotypes were compared against *in silico* predicted spoligotypes. For this comparative analysis, we used spoligotypes that had been reported for approximately 11% of our isolates from conventional lab-based spoligotyping at APHA. We also performed *in silico* spoligotyping to obtain a better understanding of the spoligotype diversity in our dataset since only a relatively small percentage of isolates had been genotyped in the lab. Seven isolates appeared to have lab-inferred spoligotypes that were not consistent with their *in silico* predicted spoligotypes. Upon closer examination, these inconsistencies appear to be most likely owing to typing errors in the lab since the lab-inferred and predicted spoligotypes differed by a single spacer, and in most cases the predicted spoligotype matched what was expected for the samples in question. Only one case was suspected to be due to mis-labelling in the lab since the lab-inferred and predicted spoligotypes differed by more than one spacers, which were not contiguous, and the lab-inferred spoligotype did not match what was expected for the sample in question.

# Supplementary Figures and Tables

## Supplementary Figures





**Supplementary Figure 1.** Maximum-likelihood phylogeny of 2,823 *Mycobacterium bovis* sequences from across Great Britain. The outgroup of the tree (*M. caprae* sequence SRR7617662) has been omitted for better visualisation. The scale bar represents units of substitutions per site. Major clades (B1-B7) are labelled on the tree and minor clades (B6-11 – B6-92) are shown next to the tips of the tree. Nodes with bootstrap support values over 95% are represented by red dots while nodes with bootstrap support values over 80% are represented by purple dots. WGS clade, lab-inferred spoligotype and *in silico* predicted spoligotype for each isolate are indicated in the bars next to the phylogeny (from left to right). Grey bars on the lab-inferred spoligotype annotations mean that the respective isolates have not been typed or failed to produce a spoligotype in the lab. Grey bars on the *in silico* predicted spoligotype annotations mean that a unique spoligotype pattern was predicted that had not been observed for any other isolate (singletons).

**Supplementary Figure 2.** Frequency distribution of pairwise SNP distances between isolates from select WGS clades. Note that the scale of the x-axis differs in each plot.

**Supplementary Figure 3.** Frequency distribution of pairwise SNP distances between isolates belonging in a single WGS clade. Note that the scale of the x-axis differs in each plot.

**
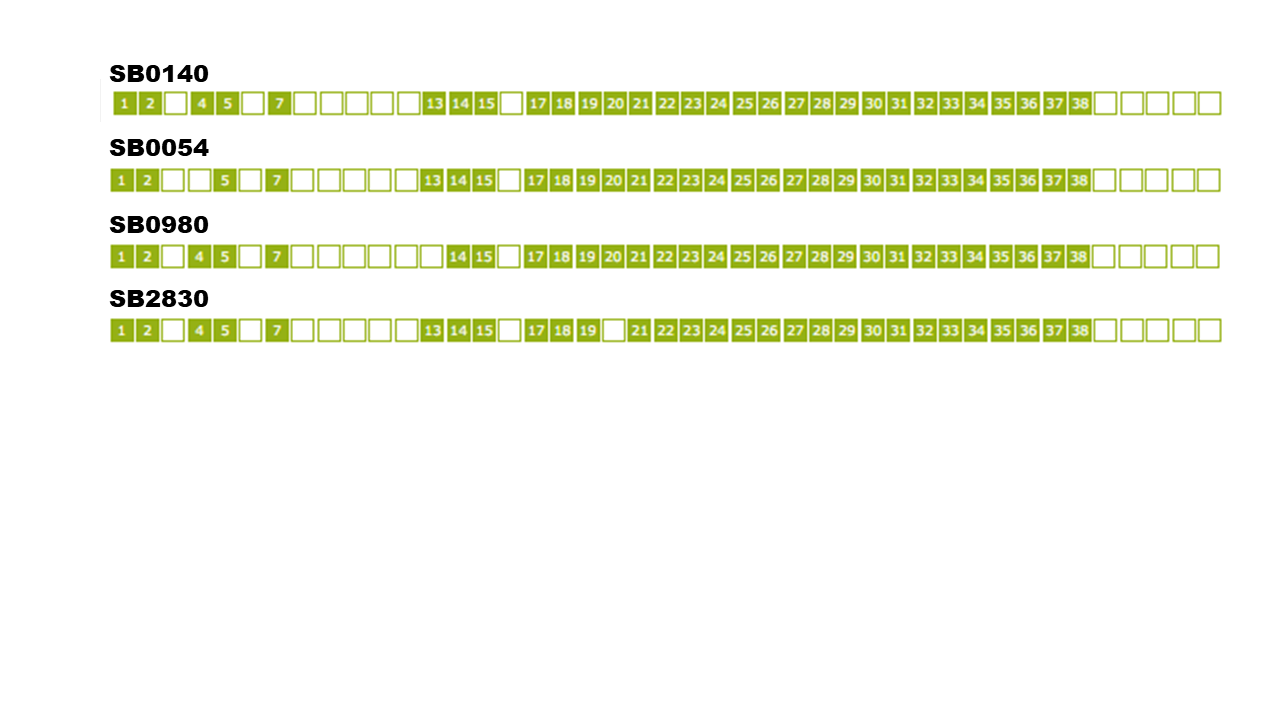
**

**Supplementary Figure 4.** Spoligotype patterns for SB0140, SB0054, SB0980 and SB2830, which are all found within WGS clade B6-87 and are closely related across their genomes despite differences in their spoligotype patterns. Filled boxes indicate presence of spacers while empty boxes indicate absence of spacers.

## Supplementary Tables

**Supplementary Table 1.** Regions of the AF2122/97 reference genome (NC_002945) that were excluded from SNP calling.

**Supplementary Table 2.**  List of sequences that were analysed to identify unique SNPs for each WGS clade.

**Supplementary Table 3.** Clade-specific SNPs and their SNPeff annotations. Columns “Element”, “Gene” and “Locus” were extracted from the annotation of the updated *M. bovis* AF2122/97 genome assembly (GCF_000195835.2). Columns “Annotation”, “Impact”, “Gene_Name” and “Gene_ID” are extracted from the SNPeff predictions. Clade-specific SNPs for B6-16 have the reference call in that clade and the alternative call in all other clades since the reference genome (AF2122/97) belongs in this clade.
